# Enhancing Cystic Fibrosis Immune Regulation

**DOI:** 10.3389/fphar.2021.573065

**Published:** 2021-05-13

**Authors:** Anna M. van Heeckeren, Morgan T. Sutton, David R. Fletcher, Craig A. Hodges, Arnold I. Caplan, Tracey L. Bonfield

**Affiliations:** ^1^ Pediatrics, Case Western Reserve University School of Medicine, Cleveland, OH, United States; ^2^ Department of Biology, Case Western Reserve University School of Medicine, Cleveland, OH, United States; ^3^ Skeletal Research Center, Case Western Reserve University School of Medicine, Cleveland, OH, United States; ^4^ National Center for Regenerative Medicine, Case Western Reserve University School of Medicine, Cleveland, OH, United States; ^5^ Departments of Genetics and Genome Sciences, Case Western Reserve University School of Medicine, Cleveland, OH, United States; ^6^ St. Jude Children’s Research Hospital Graduate School of Biomedical Sciences, Memphis, TN, United States

**Keywords:** bone marrow transplantation, hematopoietic cells, macrophages, mesenchymal stem cells, immune support, infection, inflammation, cystic fibrosis

## Abstract

In cystic fibrosis (CF), sustained infection and exuberant inflammation results in debilitating and often fatal lung disease. Advancement in CF therapeutics has provided successful treatment regimens for a variety of clinical consequences in CF; however effective means to treat the pulmonary infection and inflammation continues to be problematic. Even with the successful development of small molecule cystic fibrosis transmembrane conductance regulator (CFTR) correctors and potentiators, there is only a modest effect on established infection and inflammation in CF patients. In the pursuit of therapeutics to treat inflammation, the conundrum to address is how to overcome the inflammatory response without jeopardizing the required immunity to manage pathogens and prevent infection. The key therapeutic would have the capacity to dull the inflammatory response, while sustaining the ability to manage infections. Advances in cell-based therapy have opened up the avenue for dynamic and versatile immune interventions that may support this requirement. Cell based therapy has the capacity to augment the patient’s own ability to manage their inflammatory status while at the same time sustaining anti-pathogen immunity. The studies highlighted in this manuscript outline the potential use of cell-based therapy for CF. The data demonstrate that 1) total bone marrow aspirates containing *Cftr* sufficient hematopoietic and mesenchymal stem cells (hMSCs) provide *Cftr* deficient mice >50% improvement in survival and improved management of infection and inflammation; 2) myeloid cells can provide sufficient *Cftr* to provide pre-clinical anti-inflammatory and antimicrobial benefit; 3) hMSCs provide significant improvement in survival and management of infection and inflammation in CF; 4) the combined interaction between macrophages and hMSCs can potentially enhance anti-inflammatory and antimicrobial support through manipulating PPARγ. These data support the development of optimized cell-based therapeutics to enhance CF patient’s own immune repertoire and capacity to maintain the balance between inflammation and pathogen management.

## Introduction

The question of immune sufficiency in CF has been the focus of scrutiny for many years due to the inability to resolve bacterial infections and the overzealous inflammatory response (Bruscia and Bonfield 2016a; Ralhan et al., 2016). CF patients are inefficient at managing chronic pulmonary infections with pathogens such as *Pseudomonas aeruginosa*, *Mycobacterium abscessus*, *Mycobacterium avium*, *Aspergillus fumigatus* and *Burkholderia cepacia*, all pathogens generally found in scenarios of immune insufficiency (Knutsen and Slavin, 2011; Nichols and Chmiel, 2015; Roesch et al., 2018; Bell et al., 2019; Martiniano et al., 2019). Susceptibility to infection in CF has been associated with defective mucociliary clearance, mucus plugging, epithelial cell pro-inflammatory sensitivity and the inability for innate immune cells to reach or effectively interact with the inciting pathogens (Ma et al., 2018; Bell et al., 2019). Treatment for CF has advanced with successful CFTR modulators, inhaled saline, improved antibiotics, active elastase inhibitors, nutrition, supportive care and chest clearance techniques (Elborn, 2016; Spielberg and Clancy, 2016). Unfortunately, these advances in care do not resolve the inflammation and infection that has already been established in CF patients (McElvaney et al., 2018; West and Flume, 2018). Further, the balance between immunosuppression for chronic lung inflammation and prevention of infection have been more elusive with patients living longer and the dynamic changes associated with disease progression and shifts in the species of inciting pathogens (Clancy et al., 2019; Egan, 2020). Therapeutics that could improve the immune inefficiencies in CF have the potential to provide patients with additional gain toward management of infection and inflammation resulting in improved clinical outcomes. Finally, chronic diseases that are associated with chronic inflammation are impacted by aging and immuno-senescence (Pawelec, 2017; Fulop et al., 2018) suggesting that providing a means of refreshing CF immunity may aide in maintaining minimal morbidity and mortality until a cure for CF is ultimately achieved (Aiello et al., 2019; Bezzerri et al., 2019).

Immune dysregulation and inefficient management of infection, whether intrinsic or resultant from CF disease pathophysiology is important to understand and treat therapeutically. The CF literature has several references to therapeutic strategies focusing on specific immune cells such as: neutrophils, monocyte/macrophages, T lymphocytes, dendritic cells and airway epithelial cells (Bruscia and Bonfield, 2016a; Ralhan et al., 2016). Many of these studies have utilized the strengths of the CF knockout mouse infection model to mimic CF infection and inflammation (Soltys et al., 2002; Heeckeren et al., 2006; Bruscia et al., 2009). In CF, the cystic fibrosis transmembrane conductance regulator (*Cftr*) knockout (KO) mouse has provided important insight into avenues for immune cell based interventions with better understanding of the roles epithelial cells, macrophages and T-cells (Hodges et al., 2011; Bonfield et al., 2012; Ng et al., 2014). As is the case of most animal models of human disease, the CF mouse model does not recapitulate all aspects of human CF, however, even with these criticisms, the CF mouse has played an essential role in understanding the inflammatory response to pathogens and for the development of the other more complex CF animal models (Stotland et al., 2000; Bragonzi, 2010; Fan et al., 2018; Rosen et al., 2018; Hodges and Conlon, 2019). This manuscript will combine historical non-congenic and congenic transplantation studies along with more recent cell specific knockout models and human mesenchymal stem cells (hMSCs) to test the hypothesis that immune supportive therapy can provide clinical benefit in CF.

## Materials and Methods

### Mice

All procedures involving mice were reviewed and approved by Case Western Reserve University, Institutional Animal Care and Use Committee. *Cftr* ^*tm1Unc*^ mice were obtained from The Jackson Laboratory (Stock#002196) ((Snouwaert et al., 1992) and bred by the Case Western Reserve University Cystic Fibrosis Mouse Models Core. Creation of the conditional alleles *Cftr* ^*fl10*^ and *Cftr* ^*invfl10*^ as well as the LysMCre-control (WT) are described elsewhere (Hodges et al., 2008; Hodges et al., 2011; Bonfield et al., 2012). Genotyping of the mice was completed by PCR analysis using DNA extracts from tissue. To detect the *Cftr* ^*fl10*^ allele (408 bp) and the *KO* allele (148 bp), primers P1 (5′ GTA​GGG​GCT​CGC​TCT​TCT​TT-3′), P2 (5′-GTA​CCC​GGC​ATA​ATC​CAA​GA-3′), and P3 (5′-AGC​CCC​TCG​AGG​GAC​CTA​AT-3′) were used. To detect the *Cftr* ^*invfl10*^ allele (563 bp) and the knock-in (*KI*) allele, primers P1, P2, and P4 (5′- CAC​CCA​CTC​CAG​CTT​AAT​CC-3′) were used. PCR reactions were completed using 30 cycles of 95°C for 30 s, 55°C for 30 s, and 72°C for 30 s. The bone marrow transplantation studies were all done with B6.129P-2 *Cftr* ^*tm1Unc*^ (*Cftr* ^*tm1Unc*^, null) and the appropriate littermate controls. The different murine models are listed in Table 1.

**TABLE 1:** Nomenclature of murine models.

Genotype	Description
B6.129P2-Cftr^tm1Unc^	Cftr deficient everywhere (CF)
C57BL/6J	Cftr is expressed everywhere (WT)
Cftr^fl10^	Floxed KO control. Cftr is everywhere (like WT)
Cftr^invfl10^	Floxed KI control. Cftr is nowhere (like a cftr null, CF)
Cftr^fl10^ + LysMCre	Floxed KO everywhere but myeloid lineage (KO)
Cftr^invfl10^ + LysMCre	Floxed KI. Cftr deficient everywhere but the myeloid lineage (KI)

### Preparation of Bone Marrow Cells for Transplant Studies

Bone marrow aspirates were obtained from the femur and tibia as described previously (Bonfield et al., 2008). Bone marrow recipient mice were provided with antibiotics (sulfatrim suspension of sulfamethoxazole and trimethoprim, USP 200 mg/40 mg per 5 ml, cherry flavor; Henry Schein 4207716) in the drinking water (20 ml sulfatrim to 1 L of water; 0.8 mg/ml sulfamethoxazole and 0.016 mg/ml trimethoprim) starting two weeks before irradiation with a single dose of 8 Gy of Ce^137^ and the same day total bone marrow cells were injected into the tail vein (∼10^6^ cells/100 µl RPMI; 0.1 ml/mouse). These mice were maintained on water containing antibiotics for another 4 weeks followed by autoclaved water. The mouse strain in which bone marrow cells are harvested will be listed first, the recipient mice will be designated after the arrow (example: WT → CF, WT bone marrow aspirates were harvested and injected into CF mice).

### *Pseudomonas aeruginosa* Lung Infection

Transplanted mice were infected with *P. aeruginosa* laden agarose beads, three months after bone marrow reconstitution (van Heeckeren et al., 1997; Bonfield et al., 2012; Hsu et al., 2016). Mice were assessed clinically once daily for 3 or 10 days for coat quality, posture, ability to right themselves after being placed in lateral recumbence, ambulation and body weight utilizing a standardized clinical score profile outlined in Table 2. Post-mortem was completed during the study and at the termination on any mouse that succumbed during the study to determine cause of death.

**TABLE 2:** Key to murine model clinical outcomes.

Score	Histologic findings	Clinical scores	Gross lung pathology
0	Within normal limits	Healthy appearance and activity	Within normal limits
1	Presence of inflammatory cells	Scruffy appearance	Darker red
2	Presence of interstitial inflammation and fibrotic foci	Scruffy and dehydrated	Few nodules
3	Interstitial and alveolar inflammation, fibrosis	Scruffy, dehydrated and decreased activity	Several nodules, <25% consolidation
4	N/A	Scruffy, dehydrated and minimal activity	Numerous nodules 25–50% consolidation
5	N/A	Moribund or dead	Numerous nodules>50% consolidation

### Bronchoalveolar Lavage (BAL)

Mice were injected subcutaneously with a lethal dose of ketamine (80 mg/kg) and xylazine (10 mg/kg) (Hsu et al., 2016; Bonfield et al., 2012). The lungs were exposed followed by inserting a cannula through the trachea into the bronchi with a BAL wash of 1 × 1 ml aliquot of warm PBS. The BAL was evaluated for total and cell type (differential) cell counts with cytokine analysis. In the case of culturing the cells for gene expression, 3 × 1 ml aliquots of warm PBS were instilled in the lung.

### Bone Marrow Derived Macrophages (BMDM)

BMDM were isolated as previously described (Bonfield et al., 2008) and counted for viability (trypan blue exclusion) followed by culture for 7–10 days with L929 support medium containing macrophage colony stimulating factor.

### Cytokine Analysis

Cytokines TNF-α, IL-1β, IL-6, MIP-2 and KC, by Luminex according to the manufacturer’s recommendations (R&D Systems, Minneapolis, MN). Cytokine concentrations were normalized to units/ml of epithelial lining fluid (ELF).

### Human Mesenchymal Stem Cells (hMSCs)

Human bone marrow derived hMSCs were obtained in collaboration with Dr. Arnold Caplan’s laboratory under (IRB# 09-90-195) and validated according to stringent guidelines previously outlined (Lennon and Caplan 2006; Crapnell et al., 2013). hMSCs supernatants (containing 5% fetal bovine serum) were obtained from hMSC cell cultures that were grown in the absence of antibiotics at confluence for 72 h. Conditions using the supernatants utilized 1:1 dilution of the hMSC supernatant with the bone marrow derived medium required for appropriate growth (Sutton et al., 2017).

### Human Sputum Cell Preparations

All samples were obtained with informed consent and compliance by the Case Western Reserve University/Rainbow Babies and Children’s Hospitals IRB approval (IRB#11-67-200). CF sputum was processed and cells were obtained using standardized procedures (Matuska et al., 2016). Induced sputum was provided by the CFF Integrated Cytology Core which supports TDN trials. Controls (n = 3, HC) were obtained from healthy volunteers in the Case Western Reserve University community.

### RT-PCR

BAL, sputum BMDM or hMSCs were processed for messenger ribonucleic acid (mRNA) followed by complementary deoxyribonucleic acid (cDNA) synthesis for chemokine gene expression using RT-PCR. Quality of mRNA and cDNA was assessed through nanodrop spectrophotometry (optimal threshold 260–280 nm). Validation was done through use of a reference gene peptidyl prolyl isomerase (hPPIA) for human samples or GAPDH for mouse cells. The expression of interleukin 6 (IL-6), tumor necrosis factor alpha (TNFα), peroxisome proliferator activator receptor gamma (PPARγ) was compared to the expression of hPPIA. All PCR samples were compared to hPPIA expression for fold change in each target gene threshold cycle (dCT).

### Statistics

Statistical analysis was performed using GraphPad Prism (version 6.5–8.0). Data are shown as means ± standard deviation, unless indicated otherwise. Comparisons of survival at a specified time (e.g., 10-days) were made using Fisher’s exact test. Two-group comparisons for continuous data were made using one-way ANOVA and Student’s t-test or the Kruskal-Wallis and Wilcoxon rank sum tests. The Bonferroni correction was used when making pairwise comparisons among 3 or more groups. When pooling data from more than one experiment, the data was evaluated using a two-way ANOVA, or using the nonparametric van Elteren test with each experiment as a stratifying factor. Analyses of log or square-root transformation were utilized to compare between experimental conditions at a single point with paired t-tests and slopes over time (van Heeckeren and Schluchter, 2002). In the chronic infection models, survival curves were compared using stratified log-rank tests, cell treatment as the as the strata. Pathology (e.g., bacterial load, white blood cell counts, and cytokines) was log-transformed as necessary to compared between groups or conditions using one or two-way ANOVA, treating donors as experimental blocks. Differential counts are expressed as percentages will be transformed using logit or arcsin (square root) transformations to stabilize variances to meet normality assumptions. All significance was defined by the 95% confidence interval at *p* ≤ 0.05.

## Results

### Bone Marrow Transplantation and *Pseudomonas aeruginosa* Infection in *Cftr* Deficient Mice

**FIGURE 1:**
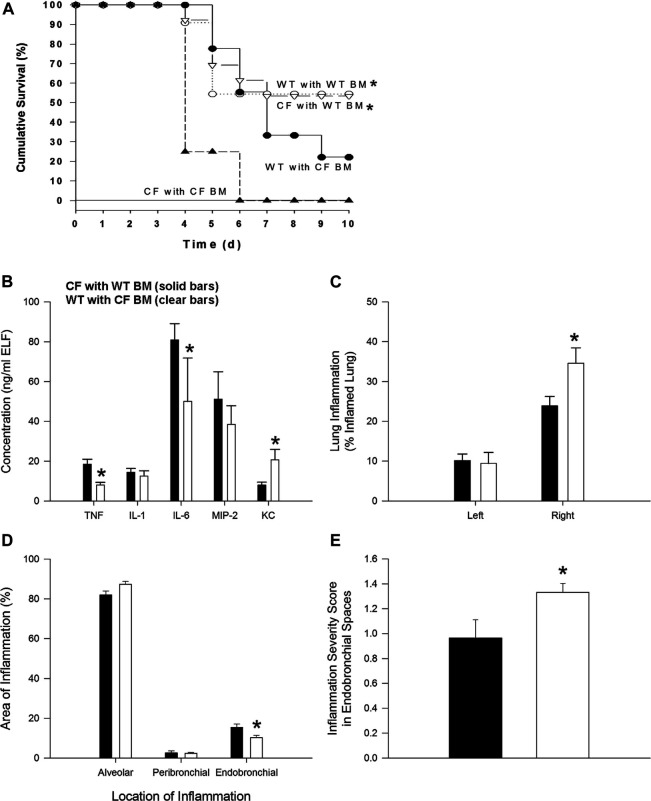
Bone marrow non-congenic chimeras and response to chronic *P. aeruginosa infection*. CF→CF (closed triangles), WT→CF (open triangles; closed bars), CF→WT (closed circles; clear bars) and WT→WT (open circles) mice were inoculated with *P. aeruginosa*-laden agarose beads on Day 0, three months following bone marrow reconstitution. Mice were monitored daily following infection. Moribund mice were euthanized for humane reasons and included as if spontaneous death had occurred. **(A)** Significantly different survival rates on Day 10 between the four possible groups (*p* = 0.03; Fisher’s exact test) where donors and recipients were males. Significantly different survival rates on Day 10 with improvement in survival of CF animals with WT BM and decrease survival of WT mice with CF BM compared to survival of CF animals and WT animals without BM four possible groups (*p* = 0.0284; Fisher’s exact test). **(B)** BAL fluid was collected from one subset of WT→CF (N = 11), CF→WT (N = 10) mice sacrificed three days after *P. aeruginosa* challenge for cytokine and chemokine analysis. *Significantly different from CF → WT mice (*p* ≤ 0.03; Wilcoxon test). **(C)** Following BAL fluid collection in mice represented in Figure 1C, the areas of inflammation in the left and right lungs were evaluated using point counting. *Significantly different from CF → WT mice (*p* ≤ 0.01; Wilcoxon test). **(D)** The area of the location of the inflammation in the right lung is shown. *Significantly different from CF → WT mice (*p* = 0.03; Wilcoxon test). **(E)** The severity of the inflammation within the endobronchial spaces of the right lung is shown. *Significantly different from CF → WT mice (*p* = 0.04; Wilcoxon test). Data in the bar graphs are shown as the means ± SEM.

**FIGURE 2:**
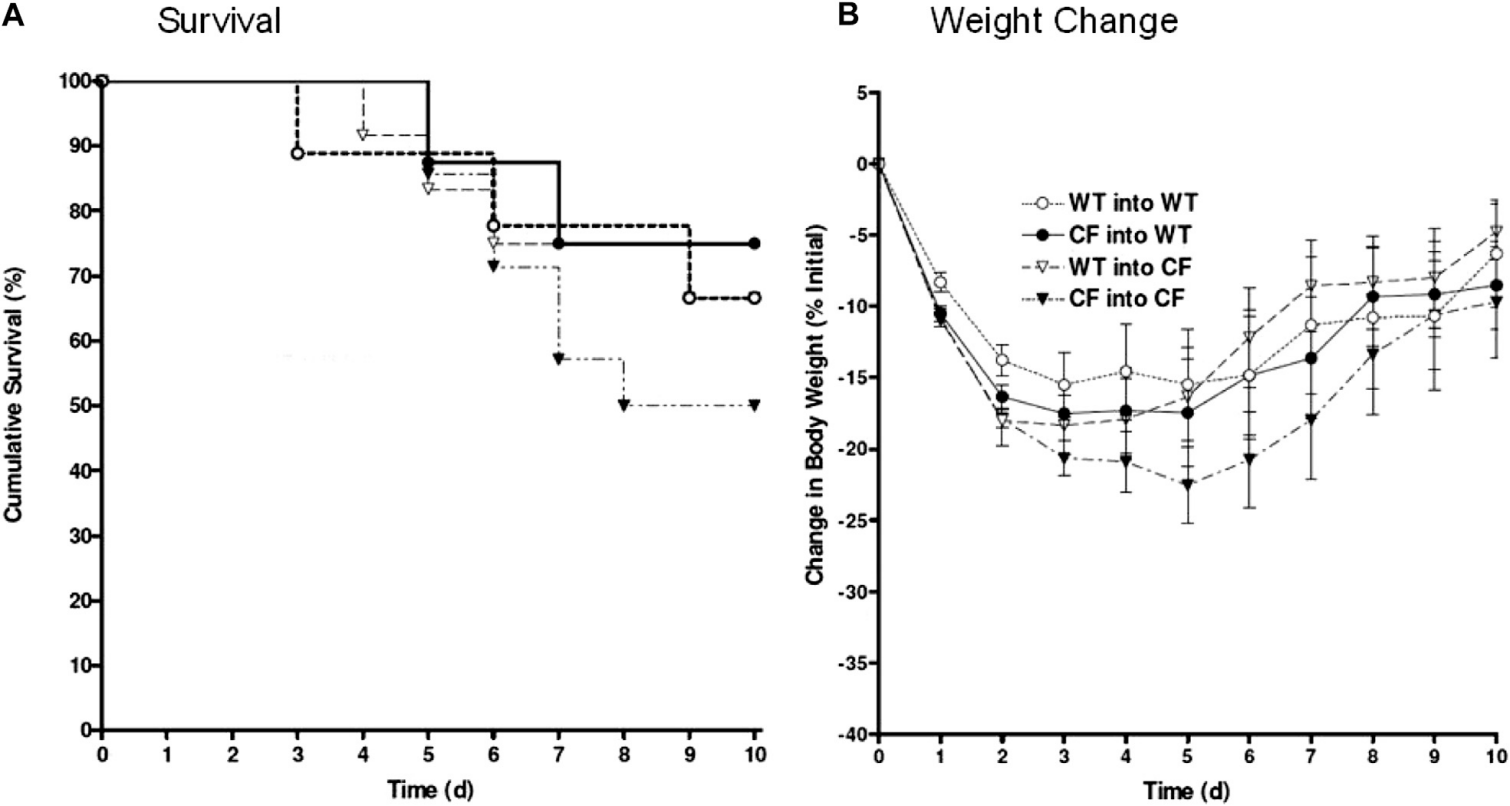
Bone marrow congenic chimeras and response to chronic *P. aeruginosa* infection. Bone marrow chimeras were generated using congenic CF (C*ftr* ^tm1Unc^) mice and litter mate controls. Mice were >6 generations on the C57Bl/6 background. Mice were irradiated and given bone marrow by retro-orbital administration. None of our animals died using this protocol suggesting efficiency of engraftment. CF mice given CF bone marrow showed the least ability to survive, averaging around 50% survival (black triangles, n = 12). C*ftr* ^*tm1Unc*^ null mice given wild type bone marrow (open triangles, n = 10) had 76% survival. WT mice given CF (closed circles, n = 12) had 80% survival. WT mice given WT bone marrow (open circles, n = 10) had 76% survival. WT mice infected without transplantation had 94 ± 6% survival compared to 50 ± 13% survival of CF mice infected without transplantation. Weight changes **(B)** are consistent survival **(A)**.

Bone marrow transplantation studies were done with non-congenic (Figure 1) and congenic (Figure 2) *Cftr* deficient (CF) mice and wild type (WT) controls. WT or CF mice were irradiated, and reconstituted with an intravenous infusion of either male or female autologous total bone marrow aspirates in sex-mismatched groups to follow the model and treatment regime. WT aspirates were infused into WT recipients (WT → WT) and CF aspirates were infused into CF recipients (CF → CF). To assess whether the irradiation and/or transplant sex mismatched procedures caused baseline changes of radiation pneumonitis or other effects, lung inflammation and survival 3 months after the transplant mice were infected with *P. aeruginosa* (average of 3.24 × 10^4^ CFU/mouse embedded into 10^3^ microns in diameter agarose beads) and followed for out to 10 days. There was no significant difference between counts of BAL inflammatory cells in the different transplant paradigms in the male to female transplants, with no statistically significant histologic inflammation scores between the groups (Supplementary Table S1A). These results suggested only minimal lung inflammatory changes due to the irradiation and transplant schemes and sex-mismatched transplant conditions (Supplementary Table S1B).

In the next series of studies, bone marrow transplant studies were conducted between the different mouse genotypes: WT → WT, CF → CF, WT → CF and CF → WT (bone marrow source → recipient). After 3 months mice were infected with agarose beads embedded with *P. aeruginosa*. There were no differences in the WT → WT and CF → CF groups, from the non-transplanted WT and CF infected mice respectively. WT → WT mice had a higher survival rate than CF → CF (55% vs. 0% respectively, *p* = 0.02). Improved survival and inflammation occurred when CF mice received WT bone marrow (WT → CF) compared to CF receiving CF (CF → CF) whereas decreased survival and inflammation occurred when WT mice received CF bone marrow (CF → WT) (Figure 1A; *p* < 0.03; logistic regression model). WT → CF mice had a survival rate indistinguishable from that of WT → WT mice (54 vs. 55% respectively), a significant improvement from the CF → CF transplant series (0% by day 6, *p* = 0.02). CF → WT mice (22% survival) had an intermediate response between CF → CF (0%) mice and WT → WT mice (55%), suggesting a CF specific hematopoietic impact on the transplantation.

To monitor changes in inflammation, BAL cytokines and cellular infiltrate types were quantified. TNF-α and IL-6 were decreased in the WT → CF (open bars) with statistically higher levels of KC compared to the CF → WT (Figure 1B, *p* < 0.05, dark bars). Absolute and differential cell counts were not significantly different between the two groups, except for the neutrophil levels (Supplementary Tables S2A,S2B). In evaluating the histological differences, there was more inflammation in the WT → CF (opened bars) than the CF → WT (dark bars). The predominance of inflammation in the right lobe is likely due to the trans-tracheal administration of the agarose beads embedded with *P. aeruginosa* is instilled in the right lobe, inducing inflammation in that area (Figure 1C). Histological evaluation demonstrated that although the endobronchial inflammation was greater in the WT → CF mice (Figures 1D,E), the total severity score of lung infection induced inflammation was higher in the CF → WT mice. This is likely due to the heterogeneity in the endo-bronchial sections and the impact of mix-matched HLA on the inflammation post-implant.

### Congenic Bone Marrow CFTR Expression Alters the Severity of the Pulmonary Response to Chronic Lung Infection with *Pseudomonas aeruginosa*

In these studies, the bone marrow chimera studies were repeated using the same transplantation combinations outlined in Figure 1 but utilizing congenic CF and WT mice. The survival kinetics and weight profile of the studies (10–12 congenic animals/group) are outlined in Figure 2. Only 50% of the congenic CF mice transplanted with CF bone marrow (CF → CF) survived whereas 66% of the CF mice transplanted with WT bone marrow (WT → CF) survived (Figure 2A). WT mice given congenic CF bone marrow (CF → WT) had 76% survival, similar to 66% survival of WT mice transplanted with WT bone marrow. Although not done at the same time due to the sheer size of the experiments, WT mice chronically infected *P. aeruginosa* without transplantation traditionally have a 94 ± 6% survival compared to 50 ± 13% survival of infected congenic CF mice, in the absence of transplantation (van Heeckeren et al., 1997; Bonfield et al., 2012; Hsu et al., 2016). The survival post-infection with and without treatment was tracked through daily weight loss kinetics (Figure 2B), which tracked with the survival.

To determine the neutrophilic response to infection in the lung post-transplantation, broncho-alveolar lavage was performed on surviving mice followed by an assessment of total cell count and cell type. Transplantation of the CF animals with CF bone marrow (CF → CF) had similar levels of neutrophils to the CF animals in the non-treated group (Table 3, 343 ± 21 vs. 330 ± 121, respectively). Reconstitution of the CF animals with WT bone marrow (WT → CF) trended toward a decreased in both absolute and relative numbers of neutrophils in the BAL (343.6 ± 20.9 to 300.2 ± 52.6 absolute neutrophils, *p* = 0.08; 69.8 ± 5.5 to 61.9 ± 4.04 relative neutrophils, *p* = 0.06). There was a 23% and 4% increase in alveolar macrophages and lymphocytes; respectively which was not significantly different between the transplant groups. Reconstitution of the WT mice with CF bone marrow (CF → WT), however, did result in a 47% increase in BAL neutrophils (113.5 ± 21.6 to 167.9 ± 41.4, *p* < 0.05) as a response to infection with *P. aeruginosa* consistent with the non-congenic studies.

**TABLE 3:** Transplantation in congenic mice.

Experiment	Neutrophils		Alveolar macrophages		Lymphocytes
	Absolute (× 10^3^)	Relative	Absolute (× 10^3^)	Relative	Absolute (^a^× 10^3^)
WT → WT (n = 6)	113 ± 22	52 ± 5	183 ± 135	38 ± 12	6 ± 2
CF → WT (n = 6)	168 ± 41	60 ± 12	171 ± 64	46 ± 4	5 ± 2
CF → CF (n = 7)	344 ± 21	70 ± 6	107 ± 18	29 ± 4	6 ± 2
WT → CF (n = 8)	300 ± 53	62 ± 4	133 ± 48	34 ± 6	12 ± 3
CF (n = 9)	331 ± 121	80 ± 4	68 ± 7	20 ± 4	1 ± 1
WT (n = 5)	67 ± 27	59 ± 8	25 ± 7	39 ± 8	2 ± 1

^a^ × 10^3^/ml of bronchoalveolar lavage fluid; ^a^in at least 3 fields of 100 cells each.

### Myeloid Knock-Out and Knock-In Models

**FIGURE 3:**
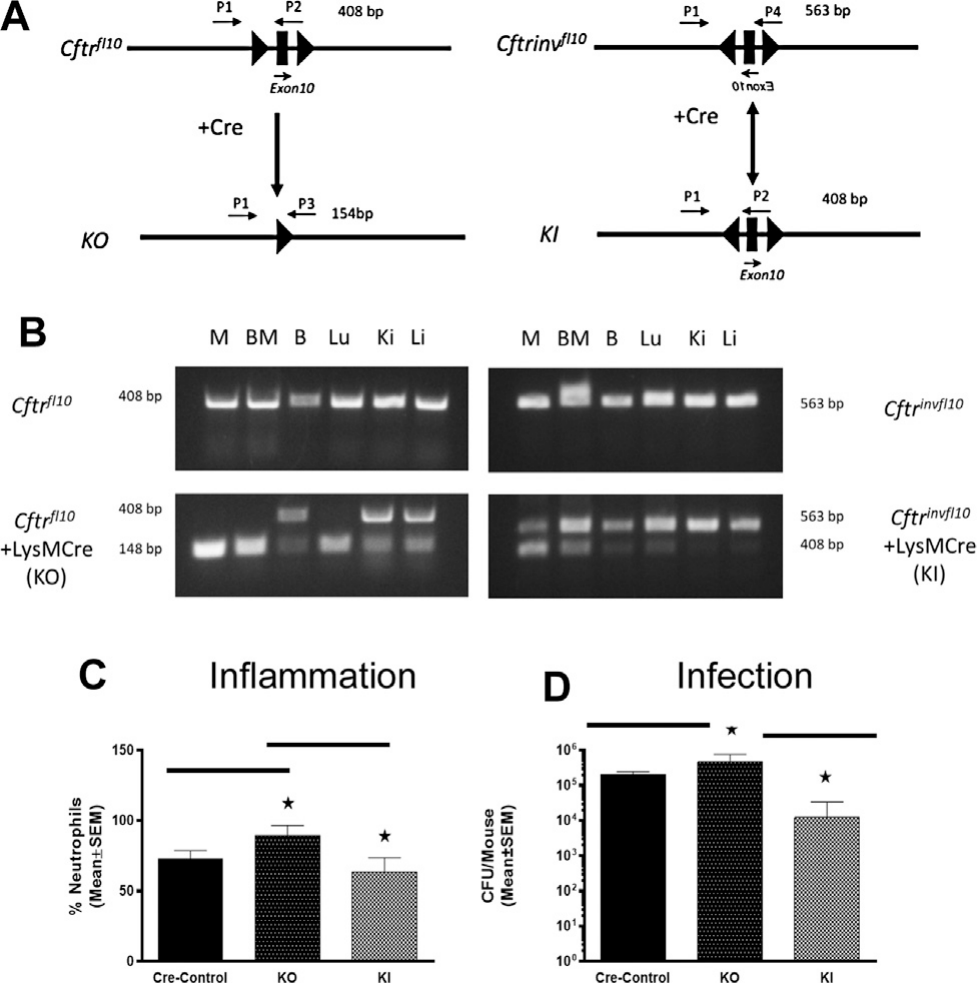
Myeloid specific *Cftr* KO and KI and response to chronic infection. **(A)** Schematic of the *Cftr* ^*fl10*^ or *Cftr* ^*invfl10*^ alleles with and without Cre recombinase present. Primers P1-P4 were used to detect the different alleles (specifics in the methods section). The *Cftr* ^*fl10*^ conversion to the KO allele is a one way reaction whereas the *Cftr* ^*invfl10*^ conversion to the KI allele is bidirectional. **(B)** DNA amplification of the region surrounding exon 10 from various tissues of mice homozygous for *Cftr* ^*fl10*^ or *Cftr* ^*invfl10*^ with and without LysMCre. Mice carrying the *Cftr* ^*fl10*^ allele display no deletion of exon 10 (408 bp) but with LysMCre display at least some of the deleted product KI (148 bp) in bone marrow derived macrophages (M), bone marrow (BM), BAL cells **(B)**, lung (Lu), kidney (Ki) and Liver (Li). Mice carrying the *Cftr* ^*invfll10*^ allele display the inverted exon 10 (563 bp) but with LysMCre display inversion of at least some of the KI allele (408 b+p) **(C,D)** Mice were infected with *P. aeruginosa* and followed for 10 days. Myeloid specific KI (n = 6) and KO (n = 6) models were compared with the WT control (n = 5) mice and each other for BAL neutrophil numbers **(C)** and *P. aeruginosa* CFUs **(D)**. The KO had significantly elevated neutrophils (*p* ≤ 0.05) and more bacteria (*p* ≤ 0.05) in the BAL than the WT control, whereas the KI model had comparable levels of neutrophils and bacteria. The KI levels of neutrophils and CFUs were significantly less than the KO model (*p* ≤ 0.05).

Murine models in which we specifically knocked-out *Cftr* in all myeloid cells had increased mortality, neutrophil recruitment and inefficient resolution of *P. aeruginosa* infection (Bonfield et al., 2012). To compliment the myeloid *Cftr* KO mice, we developed the *Cftr* knock-in (KI) model (Figure 3). The myeloid specific KO (*Cftr* ^*fl10*^ + LysM Cre,) has *Cftr* everywhere but the myeloid compartment (Table 1). The myeloid specific KI (*Cftr* ^*Invfl10*^ + LysM Cre) has *Cftr* expression in the myeloid compartment with *Cftr* deficient everywhere else. The WT (*Cftr* ^*+/+*^) mice have *Cftr* in all the tissues which express the gene. All of these mice are congenic on a C57BL/6J background. The *Cftr* allele schematic is shown in Figure 3A, demonstrating the placement of the Cre-lox sites for recombination to generate the *Cftr* KO or KI myeloid mouse models. Genotype verification of the mice is shown in Figure 3B. DNA amplification was directed toward the region surrounding exon 10 gene of *Cftr* from various tissues of mice homozygous for *Cftr* ^*fl10*^ or *Cftr* ^*invfl10*^ with and without LysMCre to generate the KO or KI. Mice carrying the *Cftr* ^*fl10*^ allele display no deletion of exon 10 (408 bp) but with LysMCre they display at least some of the deleted exon 10 product (148 bp, KO) in all tissues including bone marrow derived macrophages (M), bone marrow (BM), BAL cells (B), lung (Lu), kidney (Ki) and Liver (Li) due to the presence of myeloid cells throughout the body. Mice carrying the *Cftr* ^*invfll10*^ allele display the inverted exon 10 (563 bp) but with LysMCre display inversion of at least some the allele (408 bp, KI) leading to functional CFTR in all tissues including bone marrow derived macrophages (M) andbone marrow (BM), BAL cells (B), lung (Lu), kidney (Ki) and Liver (Li). The *Cftr* ^*fl10*^ allele can be completely converted to the deleted allele (148 bp) but due to the reversible nature of the *Cftr* ^*invfll10*^ allele the inverted allele (563 bp) will always be present and the active allele (408 bp) will never be 100%.

To investigate the response of these myeloid specific KO and KI models to infection, KO, KI and control (WT) were infected with *P. aeruginosa* embedded agarose beads and followed for 10 days. The KI mice were not different than CF mice in terms of survival, supporting the major role the epithelial *Cftr* defect plays in CF pathogenesis. However, correcting *Cftr* in myeloid cells of the CF mouse did result in improvements of some other immune responses to the *P. aeruginosa* infection. While the KO mice had an excessive neutrophilic response to infection, the KI response was significantly less than the KO approaching the WT control (Figure 3C). Further, the KI was more efficient at managing infection than the KO (Figure 3D, *p* ≤ 0.05), suggesting the prominence of the macrophage in the inefficiency of managing infections in a *Cftr* deficient environment.

### Macrophage Cell Based Therapy

**FIGURE 4:**
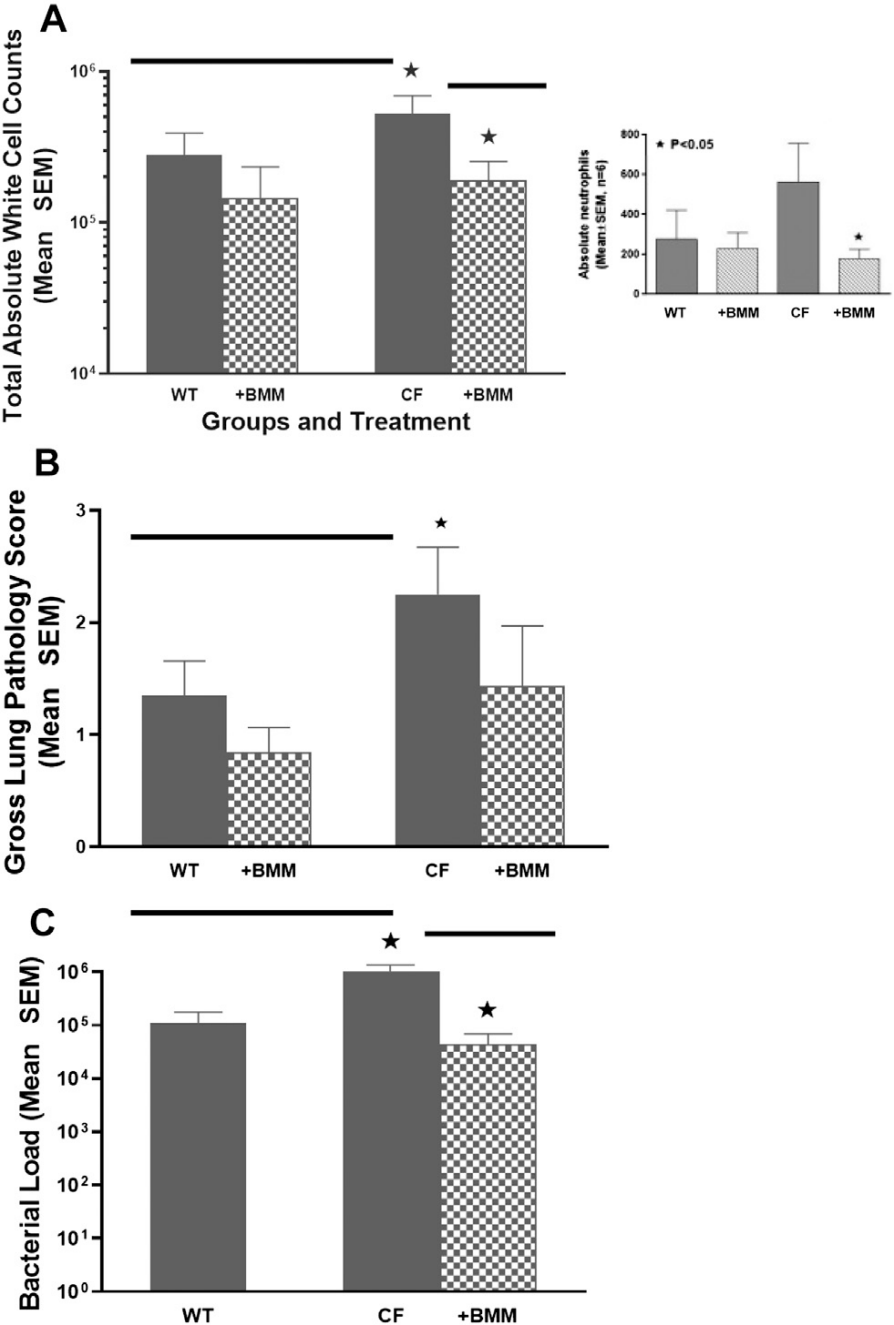
Exogenous WT BMDM decrease lung inflammation and infection *in vivo*. CF mice (n = 12) and WT controls (n = 10) were chronically infected with *P. aeruginosa* and followed for up to 10 days. Mice were infused with 10^6^ WT BMDM at day 1 post-infection. Animals were euthanized and evaluated for **(A)** white cell count, including a decrease in neutrophils (insert), **(B)** gross lung pathology and **(C)** *P. aeruginosa* infection burden. Treatment with bone marrow derived macrophages resulted in significantly (*) decreased recruitment of white blood cells improved gross lung pathology score and bacterial burden (*p* < 0.05).

Taking advantage of congenic mouse models and the ability to deliver autologous myeloid cells without immunosuppression, the next series of studies were done to evaluate the potential of providing immune support with exogenously delivered WT macrophages. In these studies, autologous 10^6^ WT BMDM were administered to either WT mice or CF mice infected 24 h previously with *P. aeruginosa* embedded agarose beads (Figure 4). Infusing WT BMDM into infected CF mice, significantly reduced the numbers of white blood cells (Figure 4A, *p* < 0.05), which includes a 30% reduction in neutrophils compared to the CF model without BMDM treatment (insert, *p* < 0.05). Treatment of the infected CF model with WT BMDM also improve lung consolidation and pathology (Figure 4B, *p* < 0.05) and the capacity to manage the *P. aeruginosa* infection (Figure 4C, *p* < 0.05). There was no major effect of the BMDM on the infected WT mice model.

### Mesenchymal Stem Cell Therapy

**FIGURE 5:**
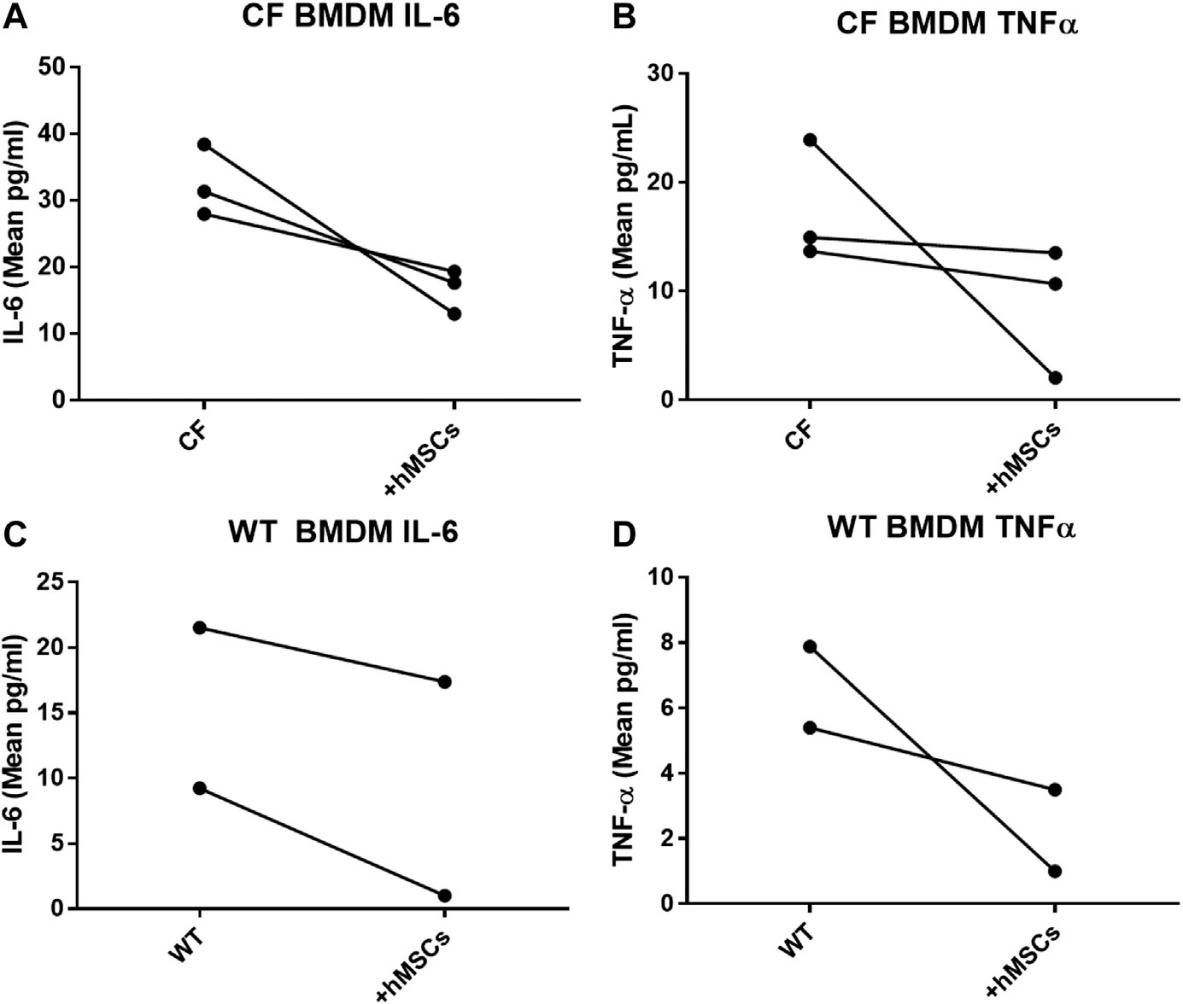
hMSCs improve BMDM Inflammatory Response to Pathogens. CF BMDM (n = 3) preparations and WT BMDM (n = 2) treated with LPS demonstrated IL-6 and TNFα secretion. This was used as to explore the suppressive capacity of three different bone marrow donor hMSC supernatants. LPS stimulated CF and WT BMDM produce both IL-6 and TNFα. Treatment of the different BMDM preparations with the different hMSCs donor preparations resulted in cumulative decreased IL-6 (*p* ≤ 0.05) and TNFα (*p* ≤ 0.05) regardless of whether the BMDM were derived from CF **(A,B)** *or* WT mice **(C,D)**. This is consistent with previously published data (Leyendecker et al., 2018).

In providing immune support, we investigated the impact of hMSCs on LPS stimulated BMDM (Figure 5). BMDM from CF (Figures 5A,B) and WT (Figures 5C,D) mice were cultured in the presence and absence of LPS to induce an inflammatory response as monitored by the secretion of TNFα and IL-6 in response to LPS. The BMDM cultures stimulated with LPS were evaluated with and without the addition of hMSC conditioned in three different studies using conditioned medium derived from 3 different donor hMSC preparations. The hMSCs conditioned medium significantly suppressed LPS induced IL-6 and TNF-α secretion relative to the LPS treated control without hMSC treatment regardless of whether the BMDM were derived from CF or WT mice (*p* ≤ 0.05, n = 3). Each of the hMSC preparations had the capacity to suppress the LPS induced IL-6 and TNFα secretion; however there was significant variability in the suppressive effect of the individual donor hMSC supernatants.

**FIGURE 6:**
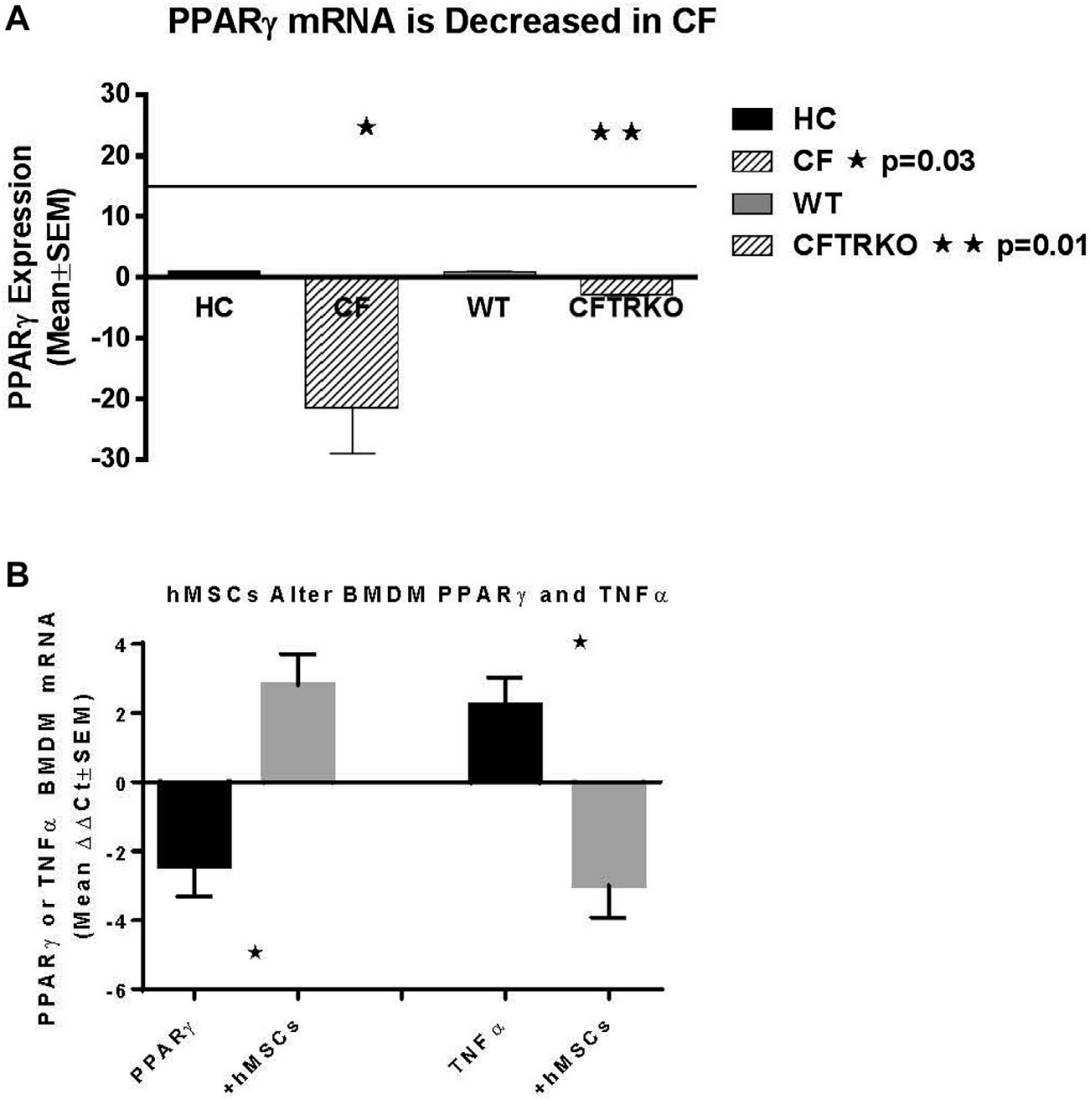
hMSC Effect on PPARγ and TNFα expression. BMDM from CF patients and CF mice were evaluated for PPARγ **(A)**. Sputum was obtained from CF patients (n = 3) and compared to healthy control (n = 3), demonstrating deficient PPARγ (*p* ≤ 0.05). BMDM from *Cftr* deficient mice also had deficient expression of PPARγ (*p* ≤ 0.05). **(B)** BMDM were stimulated with LPS and processed for PPARγ and TNFα gene expression demonstrating a decreased of PPARγ (*p* ≤ 0.05) and increased TNFα (*p* ≤ 0.05) relative to the unstiluated control of PPARγ and TNFα (2.1 ± 0.07 dCT and 1.7 ± 1.0 dCt respectively, n = 3) at baseline. The same culture conditions were done in the presence of hMSC condition medium. hMSC supernatants increased PPARγ and decreased TNFα (*p* ≤ 0.05) above the baseline controls.

Since hMSCs have the ability to facilitate the changes in the LPS induced BMDM IL-6 and TNFα production, the next series of studies explored the impact of the hMSCs on macrophage IL-6 and TNFα gene expression. Peroxisome proliferator activator receptor gamma (PPARγ) is an important regulator of the macrophage pro-inflammatory responses to infection regulating TNFα and IL-6 production (Knethen and Brune, 2002; Korbecki et al., 2019). PPARγ is deficient in CF patient sputum and BMDM from *Cftr* deficient mice exposed to LPS compared to healthy controls (HC) (Figure 6A, *p* ≤ 0.05 for sputum and *Cftr* deficient BAL cells). hMSC supernatants can alter the BMDM activity by recovering PPARγ expression decreasing TNFα expression (Figure 6B, *p* ≤ 0.05).

## Discussion

In the past 20 years, improved CF clinical care and innovation in therapeutic development has significantly improved the quality and duration of life for the majority of CF patients. However, the main cause of morbidity and mortality in CF continues to be the chronic pulmonary infection and the associated on-going inflammation (Elborn, 2016; Ma et al., 2018). In addition, there continues to be a subset of patients which are not eligible for modulator therapy either due to their specific CFTR mutation or relative intolerance to the drugs (Bell et al., 2019; Clancy et al., 2019; Egan, 2020). CF patients with established inflammation and infection, individuals not able to benefit from small molecule modulator therapy, would benefit from supportive immune therapy to enhance control over the miss-matched inflammation/infection conundrum in CF. Further, immune support would also be beneficial when considering the longer life expectancy and sustainability for patients, which become complicated with pathogen resistance, and immune-senescence (Fischer et al., 2013; Bezzerri et al., 2019).

The studies in this manuscript describe the benefit of *Cftr* sufficient bone marrow aspirates, bone marrow derived macrophages (BMDM) and bone marrow derived hMSCs in providing immune support in CF. The data highlight how supplementation of *Cftr* deficient murine models with WT total bone marrow aspirates, WT BMDM or hMSCs improved pathogen and inflammation resolution. The models also demonstrated that the WT cell-based products provided improvements in managing weight loss, survival, and lung neutrophil recruitment and cytokine profiles. The non-congenic studies demonstrate the therapeutic potency of providing CFTR sufficient hematopoietic/mesenchymal sources. The congenic studies implicate the concept of bone marrow corrective technology to boost the capacity to regulate the response to infection and management of inflammation. The development of the myeloid specific KO and KI mouse models demonstrates that hematopoietic compartment plays an essential role in managing the host immune response in CF, and promotes the idea of hematopoietic correction using CRISPR/Cas9, Talen’s or zinc fingers (Phang et al., 2013; Marangi and Pistritto, 2018; Cabrini, 2019).

The question remains as to the nature of macrophages in CF management of both infection and the host response. The macrophage is an important contributor to how the inflammatory response is initiated, sustained and resolved. Macrophages also have considerable plasticity, which could be enhanced toward targeted therapeutic impact in specific clinical settings of inflammation with or without the presence of infecting pathogens (Morales-Nebreda et al., 2015; Funes et al., 2018; Tarique et al., 2018). Macrophages are highly sensitive to their environment resulting in subtle changes in membrane proteins, which can shift their function (Bonfield, 2015; Bruscia and Bonfield, 2016b). These observations become important in the era of modulator therapy, which provides significant clinical benefit to patients who are eligible and responsive to treatment, including improved macrophage function (Zhang et al., 2018; Clancy et al., 2019; Hisert et al., 2020). Even with the modulator benefits, patients continue to have concurrent issues with infection and inflammation, as well as battles with the residual lung damage that has occurred during the pre-modulator phase of therapeutic accessibility (Burgener and Moss, 2018). The tissue specific contribution of macrophages in the context of CF is evident by the literature associated with functional contribution to CF pathophysiology (Worgall et al., 2002; Assani et al., 2017; Di Pietro et al., 2017). Currently, modulator therapy aids in the management of CFTR dysfunction in greater than 90% of patients with CF, leaving 10% of patients that will continue to struggle with the disease while sustainable treatment options are pursued (McElvaney et al., 2018; Egan, 2020). With the advent of hematopoietic supplementation, it could be that early support with autologous-based therapeutics might provide enough added immune support to minimize the pulmonary damage associated with chronic exposure to pathogens and the inflammatory response. To continue to pursue this line of therapeutic support, future studies are needed to interrogate lung macrophage phenotype relative to CF infection and inflammation and to discern the direct mechanisms for the therapeutic benefit highlighted in this manuscript.

A major caveat for macrophage based therapy is the inability to utilize the patient’s own cells (Canan et al., 2014; Stahl and Brown, 2015; Morgan et al., 2018) and the potential for graft vs. host disease (GvHD) with allogeneic sources requiring immunosuppression (Bashyam, 2007). The era of gene editing technology has provided the opportunity to consider developing corrected autologous patient macrophages for BMDM delivery. The potential of this technology is in its infancy is not ready for clinical application in scenarios like CF, especially since BMDM corrective therapy would not provide a curative outcome. Further, it would be essential to discern any changes in BMDM function or off target effects that might occur with gene editing technology (Papasavva et al., 2019). Further, one of the major hurdles in implementing gene correction is the efficiency of site directed editing in scenarios of low levels of expression of CFTR in hematopoietic cells (Abdulrahman et al., 2011; Zhang et al., 2018). Whole bone marrow aspirates, hMSC and *Cftr* sufficient macrophages provide clear implications for therapeutic development of immune support in managing CF lung infection and inflammation based upon the data we have presented in this manuscript. These observations are consistent with previous studies that have also pursued immune supportive therapeutic directives (Weiss, 2008; Bruscia et al., 2009; Bonfield et al., 2012; Sutton et al., 2017; Duchesneau et al., 2020; Zhang et al., 2020). Follow-up studies will focus on delivering CF macrophages or hMSCs in the preclinical model and further investigate the functional insufficiency of CF derived cells. The goal will be to determine the efficiencyof the CF origin of the autologous cells on the CF like manifestation observed in the murine model of the CF lung infection and inflammation-like pathophysiology. A future study will be delivering the myeloid specific Cftr KO and KI macrophages to either WT or CF mice, which again will continue to define how macrophages can be utilized to improve the care of patients with CF. Many of these studies could then be translated to more expensive complex models like the ferret or pig, to determine the effect of the cell therapy on other aspects of CF pathophysiology that are not bridged by the mouse models.

**FIGURE 7:**
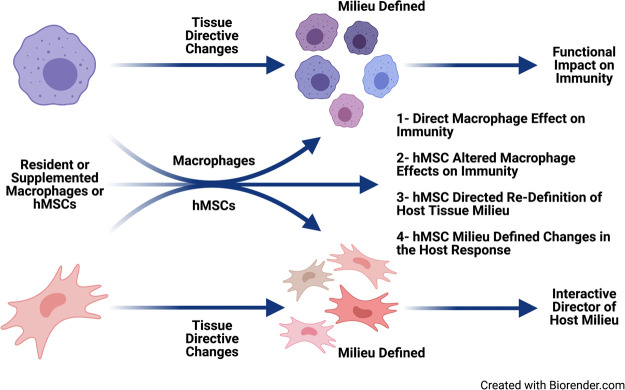
Immune supportive therapy model for cystic fibrosis. Patients with CF early become infected with pathogens which contributed to the triad of infection → inflammation → lung-damage. The lung damage continues to create a pulmonary milieu that is susceptible to infection, so the triad continues resulting in the vicious circle of that is pathologic in CF. The feasibility of providing immune support focusing on hMSCs and BMDM which could be harnessed to skew the balance of the host response to infection and establishing chronic inflammation. Macrophages and hMSCs both have their potential roles in providing clinical efficacy and potency, but the key is likely how they interact *in vivo*. Macrophages defining the hMSC phenotype due to the milieu elicited and the contribution of the functionally tissue cued hMSCs to support the resolution toward homeostasis and tissue recovery. The hematopoietic approach would minimize the CFTR induced damage to the lung and other tissue, while at the same time promoting the patient’s management of their internal milieu. CRISPR/Cas9, the advent of iPSC cells and other gene editing technologies opens the door toward the potential adding corrective immune support in CF.

hMSCs are immune evasive therefore having greater versatility over gene corrective BMDM (Auletta et al., 2015; Leyendecker et al., 2018). hMSCs can be delivered as an allogeneic source with patients not requiring immunosuppression to prevent GvHD (Auletta et al., 2015; Leyendecker et al., 2018). hMSCs treatment has been the foundation of cell-based therapeutic approaches to inflammation in a variety of diseases globally without adverse reaction, and with significant clinical response (Caplan 2017; Leyendecker et al., 2018). We have shown *in vitro*, *in vivo* and *ex vivo* clinical models that hMSCs can provide therapeutic benefit in CF through attenuating inflammation and aiding in infection resolution similar to the efficiency of macrophages (Bonfield et al., 2013). Further, we have published that hMSC treatment is antibiotic “saving” through their antimicrobial potency decreasing the required dose of antibiotics for eradication of bacteria (Bonfield et al., 2013; Sutton et al., 2016). In our “First in CF” Phase I clinical trial we have also been able to demonstrate their safety (Roesch et al., 2019). hMSCs can alter macrophage response to pathogens enabling better control of the overactive inflammatory response to infection (Figure 7), consistent. Cell-based approaches have the capacity to enhance current therapeutic availability strategies for CF through aiding the patient’s own immune capacity to regulate the inflammatory response and exposure to pathogens. CF patients are living longer,and, whether due to the chronicity of their disease or intrinsic pathophysiology, immune-senescence is likely (Pawelec, 2017; Fulop et al., 2018). Immune support may be ideal in the prevention of immune-senescence.

The concept of cell therapy for the treatment of CF lung infection and inflammation is complex, particularly with the dynamic nature of the “drug” in the form of macrophages, hMSCs or both (Hodges and Conlon, 2019). Fortunately, cell-based approaches have been described in other diseases such as alveolar proteinosis (Suzuki et al., 2014), Niemen Pick disease (Schuchman and Desnick, 2017) acute respiratory distress syndrome (Horie et al., 2018), interstitial pulmonary fibrosis (Bonfield and Caplan, 2010), bronchopulmonary dysplasia (Cerny et al., 2008), rheumatoid arthritis (Jorgensen, 2010) and others (Lachmann et al., 2014; Morrison et al., 2017; Khoury et al., 2020; Verma et al., 2020). Bone marrow supplementation or hMSC therapy is not corrective longitudinally, but would enhance the capacity of patients to manage their own immune environment. Supporting CFTR corrective approaches and managing more difficult mutations and other systemic pathologies associated with CF disease would be major benefactors of hMSCs treatment and clinical care. The immune evasiveness of hMSCs has been demonstrated in greater than 900 clinical trials currently on-going globally with no major adverse effects (clinicaltrials.gov). Many patients in these trials have effectively tolerated and benefited from multiple infusions which speaks to the safety as well as the capacity to have the option for repetitive care during exacerbations of disease (Murphy et al., 2013; Caplan, 2018).

Another benefit in hMSC cell based therapy is related to the impact hMSCs have on macrophages (Al-Rubaie et al., 2018; Leyendecker et al., 2018). hMSCs are engulfed by macrophages (*in vitro* and *in vivo*), resulting in a change in the macrophage phenotype (Morrison et al., 2017; Yin et al., 2017; Philipp et al., 2018; Harrell et al., 2019). It might be that treating CF patients with hMSCs will have a direct hMSC effect (anti-inflammatory and antimicrobial) but also an indirect effect on optimizing macrophage immune function. These studies still need to be vetted, but provide support for hMSC therapeutics as an option of supplemental care in CF. The added advantage of hMSC enhancement of antibiotic potency is also very attractive in patients who constantly suffer from chronic colonization with bacteria (Bonfield et al., 2013). hMSCs would not be a stand-alone therapy in CF, but would be given in the context of on-going treatment such as modulators, antibiotics, anti-oxidants, anti-mucolytic and the whole host of other drugs available for patient treatment schemes. These treatment algorithms would not require pre-treatment of the hMSCs, but would be considered a co-therapeutic with the patient’s traditional clinical regimen. Manipulating either macrophages or hMSCs for optimization strategies is complex but would bridge manipulated cell therapy for the unique application in CF. The capacity for manipulated cell-based therapeutic approaches is a bit more tenuous to get FDA approval, requiring innovative ways to demonstrate the safety and efficacy in the setting of CF (Philipp et al., 2018; Saeedi et al., 2019). An important aspect of hMSC therapy should focus on hMSC product development and the assurance of the optimal antimicrobial and anti-inflammatory potency in scenarios of pathogens consistent with CF infection. The concept of choosing the right hMSC donor preparation has the potential to enhance the beneficial response of the immune supportive therapy, which is also the foundation of much of our research focus currently (Caplan 2018; Sutton and Bonfield 2014; Bonfield et al., 2019).

The advancement of small molecule correctors and potentiators has made substantial contributions to the management of CF lung disease and extending patient survival (Bell et al., 2019; Clancy et al., 2019; Egan 2020). The potentiators and correctors appear to also limit the degree of insidious inflammation associated with CF lung disease; however, the success of this aspect is probably years out for defining the ultimate clinical impact on disease. As with all chronic inflammatory diseases, immunity can be impacted by chronicity of disease and the contribution of immune dysregulation based upon sustained inflammatory sequelae (Walsh, 2006; Zuo et al., 2019). The initiation of the inflammation/infection vicious circle and the inability to “turn-off” established inflammation will continue to be an issue in the foreseeable future until a cure is assured. The modulators are not curative for CF, they address the need for functional CFTR, but it is still pharmacological and short-lived, requiring a daily regimen for sustained optimal physiological dosed over time. The long-term impact and effectiveness of the small molecule drugs remains to be determined necessitating the continued pursuit of other treatment modalities. Immune support has the capacity to enhance the duration and sustainability of current therapeutics by providing patients with an extra boost to manage their own immunity. Cell based immune therapies are not curative in CF, implicating that to sustained benefit patients might require a couple doses a year (Murphy et al., 2013; Caplan, 2018). Harnessing hematopoietic stores for “efficient” management of inflammation is not a difficult vision, given the success in other diseases (Horwitz et al., 2001; Loebinger et al., 2008; Doerschuk, 2015; Sallese et al., 2017). The age of personalized medicine, hMSCs, cell based hematopoietic support, and immune correction is an opportunity to minimize disease and improve the overall health of CF patients as the pursuit of a cure for all patients with the disease is sought.

## Data Availability

The raw data supporting the conclusions of this article will be made available by the authors.
