# Supplementary material for: Enhancing Cystic Fibrosis Immune Regulation
Source: Front Pharmacol. 2021 May 13;12:573065. doi: 10.3389/fphar.2021.573065 (PMC8155373; doi:10.3389/fphar.2021.573065)
Supplement: Supplementary file 1 [file DataSheet1.pdf]

Supplemental Data

Table S1A: Survival Data for Transplant Efficiency and Sex Differences.

Table S1B: Histological Point-Counting for Inflammation on Sex Differences in the Transplant Process.

Table S2A: BAL fluid cell numbers three months following irradiation and bone marrow reconstitution using female donors into male recipients.

Table S2B: BAL fluid cell numbers three months following irradiation and bone marrow reconstitution using male donors into female recipients

Supplemental Table S1A. Survival Rate of Female and Male Transplantation Efficiency

Experiment ID	BM donor	BM recipient (male)	Survival rate (N)*
1	Female B6	B6	66.7% (6)
2	Female mix-CF	B6	70.0% (30)
3	Female mix-CF	B6	88.5% (26)
4	Female mix-CF	B6	83.3% (6)
5	Female B6	mix-CF	80.0% (30)
6	Female B6	mix-CF	75.0% (24)
7	Female B6	mix-CF	100.0% (6)
8	Female mix-CF	mix-CF	100.0% (6)
9	Male B6	B6	73.3% (15)
10	Male B6	B6	66.7% (9)
11	Male mix-CF	B6	91.7% (12)
12	Male mix-CF	B6	50.0% (6)
13	Male B6	mix-CF	86.7% (15)
14	Male B6	mix-CF	87.5% (8)
15	Male mix-CF	mix-CF	64.3% (14)
16	Male mix-CF	mix-CF	87.5% (8)

*Logistic regression model was used to test the effect of group on survival while adjusting for experiment, which was not significant for female or male donors (P = 0.96 and 0.59, respectively)

Table S1B. Lung Inflammation Score

BM Donor	BM recipient	N	Left lung	Right lung
female CF	male CF	6	1.0 ± 0.0	1.2 ± 0.4
female B6	male CF	6	1.2 ± 1.2	0.7 ± 0.5
female CF	male B6	5	0.8 ± 0.8	1.2 ± 0.8
female B6	male B6	4	0.3 ± 0.5	0.8 ± 0.5

BM = bone marrow

There were no significant differences in lung inflammation between the groups (Kruskal-Wallis test; P > 0.05)

Table S2A. BAL Leukocyte and Cell Types Post-Transplantation (Female Donors/Male Recipients).

Group<br>N	B6 → B6<br>4	mix.CF → B6<br>5	B6 → mix.CF<br>6	mix.CF → mix-CF<br>6
10^3 Leukocytes/ml	46 ± 13	78 ± 40	155 ± 139	118 ± 96
10^3 AM/ml	42 ± 11	71 ± 36	143 ± 123	110 ± 93
10^3 PMN/ml	1 ± 1	1 ± 1	1 ± 1	4 ± 3
10^3 Lymph/ml	2 ± 3	4 ± 5	11 ± 18	4 ± 2
%AM	93 ± 5	92 ± 3	94 ± 4	92 ± 4
%PMN*	2 ± 1	1 ± 1	1 ± 1†	4 ± 3
%Lymph	6 ± 4	7 ± 3	6 ± 4	5 ± 3

AM = alveolar macrophages
PMN = polymorphonuclear neutrophils
Lymph = lymphocytes
*Significantly different among the four groups using the exact Kruskal-Wallis test (P = 0.0163)
†Significantly different from mix.CF → mix-CF mice (P = 0.0077) using the Wilcoxon rank sum test when making pairwise comparisons

Table S2B. BAL Leukocytes and Cell Types Post- Transplantation (Male Donor/Female Recipient)

Group<br>N	B6 → B6<br>6	mix-CF → B6<br>3	B6 → mix-CF<br>7	mix-CF → mix-CF<br>7
10^3 Leukocytes/ml	49 ± 13	52 ± 31	49 ± 14	70 ± 30
10^3 AM/ml	41 ± 7	49 ± 8	46 ± 14	64 ± 32
10^3 PMN/ml	1 ± 1	1 ± 1	1 ± 1	5 ± 4
10^3 Lymph/ml	8 ± 8	3 ± 2	1 ± 1	1 ± 2
%AM	85 ± 12	94 ± 2	95 ± 2	89 ± 8
%PMN	2 ± 2	2 ± 1	2 ± 2	9 ± 8
%Lymph*	13 ± 12	5 ± 1	3 ± 2	2 ± 2

AM = alveolar macrophages
PMN = polymorphonuclear neutrophils
Lymph = lymphocytes
*Significantly different among the four groups using the exact Kruskal-Wallis test (P = 0.0168), but not significantly different when making pairwise comparisons using the Wilcoxon rank sum test where P = 0.0083 was considered significant.
